# Tumor metastasis: Mechanistic insights and therapeutic interventions

**DOI:** 10.1002/mco2.100

**Published:** 2021-12-02

**Authors:** Mengmeng Liu, Jing Yang, Bushu Xu, Xing Zhang

**Affiliations:** ^1^ Melanoma and Sarcoma Medical Oncology Unit State Key Laboratory of Oncology in South China Collaborative Innovation Center for Cancer Medicine Sun Yat‐sen University Cancer Center Guangzhou China; ^2^ State Key Laboratory of Oncology in South China Collaborative Innovation Center for Cancer Medicine Sun Yat‐sen University Cancer Center Guangzhou China

**Keywords:** cancer, epithelial‐mesenchymal transition, immunotherapy, metastasis, targeted therapy, tumor microenvironment

## Abstract

Cancer metastasis is responsible for the vast majority of cancer‐related deaths worldwide. In contrast to numerous discoveries that reveal the detailed mechanisms leading to the formation of the primary tumor, the biological underpinnings of the metastatic disease remain poorly understood. Cancer metastasis is a complex process in which cancer cells escape from the primary tumor, settle, and grow at other parts of the body. Epithelial‐mesenchymal transition and anoikis resistance of tumor cells are the main forces to promote metastasis, and multiple components in the tumor microenvironment and their complicated crosstalk with cancer cells are closely involved in distant metastasis. In addition to the three cornerstones of tumor treatment, surgery, chemotherapy, and radiotherapy, novel treatment approaches including targeted therapy and immunotherapy have been established in patients with metastatic cancer. Although the cancer survival rate has been greatly improved over the years, it is still far from satisfactory. In this review, we provided an overview of the metastasis process, summarized the cellular and molecular mechanisms involved in the dissemination and distant metastasis of cancer cells, and reviewed the important advances in interventions for cancer metastasis.

## Graphical abstract

Cancer metastasis is a complex process in which cancer cells escape from the primary tumor, settle and grow at other parts of the body. A variety of stromal cells, immune cells and other molecular components surrounding the tumor provide signals that enhance the metastatic potential of cancer cells. With the deepening understanding of mechanisms of tumorigenesis and metastasis in recent years, a plethora of treatment approaches have been established in patients with metastatic cancer.

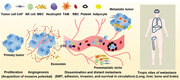

## 1 INTRODUCTION

Cancer is one of the main diseases threatening human health. According to the Global Cancer Statistics, 2020, there were 18.1 million new cancer cases and 9.6 million cancer‐related deaths worldwide.^1^ Despite the continuous development of medical technology, distant metastasis has already appeared at the time of diagnosis for many patients. Furthermore, a large number of cancer patients, both early‐ and late‐stage, may eventually develop metastatic diseases.^2^ Metastatic lesions in distant organs are difficult to be cured by current therapeutic approaches, and metastasis accounts for about 90% of death in cancer patients.^3^ In other words, the overwhelming problem highlighted by the cancer‐associated deaths is, for the most part, metastatic cancer. In order to develop the methods for preventing or treating metastasis, it is inevitable to understand the cellular and molecular mechanisms of metastasis.

In contrast to numerous discoveries that reveal the detailed mechanisms leading to the formation of the primary tumor, research on metastatic cancer is still lagging behind. Stephen Paget and James Ewing proposed the “seed and soils” and the “mechanical metastasis” hypothesis, respectively, which laid the foundation for the study of tumor metastasis.^4^, ^5^, ^6^ Cancer metastasis is a complicated process, which is regulated by various signaling pathways and modulated by the surrounding extracellular matrix (ECM). Tumor cells spread from the primary tumor mass to distant organs through blood vessels, lymphatic vessels, and transcoelomic routes.^7^, ^8^ In order to successfully metastasize from the prior site, tumor cells need to go through the following stages: local invasion, intravasation, survival in circulation, extravasation, and colonization.^9^ Furthermore, multiple components in the tumor microenvironment (TME), including immune cells, stromal cells, chemokines, and cytokines are involved in a complex crosstalk with tumor cells that affects tumor growth and metastasis.^10^, ^11^, ^12^ Surgery, chemotherapy, and radiotherapy are the three cornerstones of cancer treatment. With the deepening understanding of mechanisms of tumorigenesis and metastasis in recent years, a plethora of treatment approaches, including targeted therapy and immunotherapy have been established.^13^, ^14^, ^15^ However, the underlying mechanisms of cancer metastasis are not yet fully understood, and the strategies for preventing and inhibiting cancer metastasis are also limited.

Here, we provided an overview of the metastasis process, summarized the mechanisms underlying the dissemination and distant metastasis of cancer cells, and reviewed the important advances in interventions targeting cancer metastasis.

## 2 COMPONENTS AND MECHANISMS INVOLVED IN METASTASIS

Tumor metastasis is one of the hallmarks of tumor malignancy and one of the causes of tumor‐related death.^16^ The malignant behavior of tumor metastasis is mainly related to the malignant degree of the primary tumor, but one of the common features is that all metastases need to go through a cascade called “invasion‐metastasis cascade.”^17^ The metastasis process begins when the tumor cells gain invasiveness and lose their adhesion to the surrounding matrix including basement membrane (BM) and ECM; tumor cells migrate out of the primary tumor and invade surrounding tissues.^18^

**FIGURE 1:**
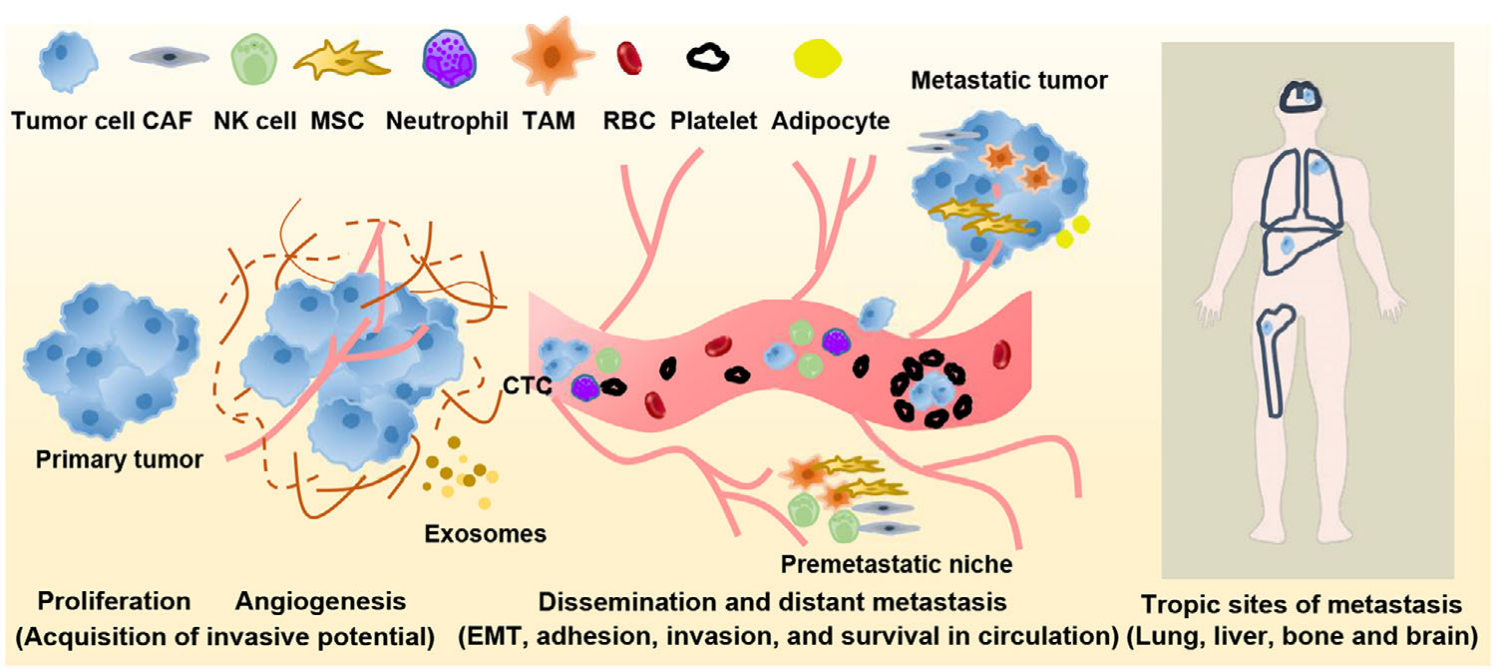
Overview of the metastatic cascade. Carcinoma cells escaping from the primary tumor migrate and invade through the basement membrane and extracellular matrix, enter the blood or lymphatic vessels, intravasate into the circulation, penetrate the blood or lymphatic vessels (extravasation), and adhere and grow in secondary sites. A variety of stromal cells, immune cells, and other molecular components surrounding the tumor provide signals that enhance the metastatic potential of cancer cells. Platelets and neutrophils can protect tumor cells by providing physical protection against shear stress, secreting mediators (such as transforming growth factor‐beta (TGF‐β)), neutralizing the cytotoxicity of NK cells and favoring immune escape. Abbreviations: BM, basement membrane; CAF, cancer‐associated fibroblast; CTC, circulating tumor cell; ECM, extracellular matrix; EMT, epithelial‐to‐mesenchymal transition; MSC, mesenchymal stem cell; NK cell, natural killer cell; RBC, red blood cell; TAM, tumor‐associated macrophage

Subsequently, disseminated tumor cells penetrate blood vessels or lymph vessels into the circulation and respond to various resistance conditions such as shear force, anoikis, and immune surveillance in the circulation.^19^ Only a small percentage of tumor cells can survive in the harsh conditions of circulation. After successfully entering the secondary site, tumor cells adhere to the endothelium of the target organ, and exudate and migrate into the organ parenchyma, which is the “pre‐metastatic niche.”^20^, ^21^, ^22^ They either enter a long‐term dormant state in the form of a single cell, or enter micrometastasis in the form of multiple cells, and finally begin to grow continuously to form clinical metastases (Figure 1).^23^, ^24^

At the beginning of the whole metastasis process, the most important thing is that there are a group of invasive and plastic tumor cells in the cancer nest, which are collectively referred to as metastasis initiating cells.^25^ Cancer stem cells (CSCs) have been proved to have the above characteristics and play a crucial role in tumor metastasis.^26^, ^27^ In addition to the role of the tumor itself, metastasis is induced by various factors, including genetic alation, abnormal epigenetic modifications, immune escape, and changes in the growth environment.^9^ Here, we will describe in detail the major factors and mechanisms involved in the metastasis cascade.

### 2.1 Key driver of metastasis: Epithelial‐mesenchymal transition (EMT)

**FIGURE 2:**
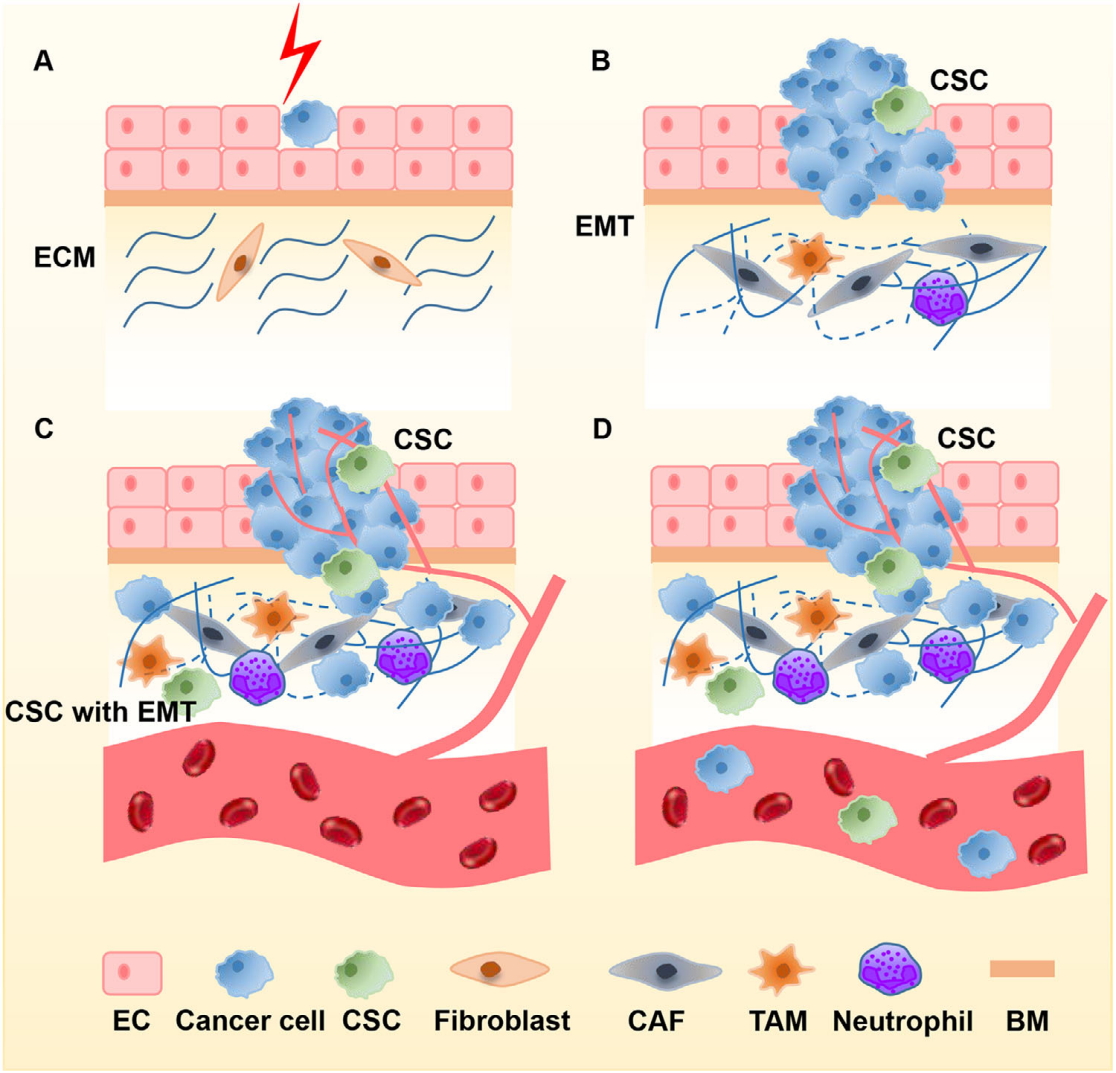
Cancer cells undergo EMT and invade into circulation. (A) A single transformed epithelial cell remains quiescent for a period of time. (B, C). The transformed cells proliferate and generate a small intraepithelial colony, accompanied by the formation of cancer stem cells. Cancer cells destroy the basement membrane, undergo EMT, and migrate and invade through the basement membrane and extracellular matrix. Normal extracellular matrix undergoes cancer‐associated remodel. Meanwhile, cells and molecular components in tumor microenvironment (TME; CAFs, TAMs, neutrophils, MSCs…) surrounding the primary tumor enhance cancer cell survival, proliferation and metastasis. (D) Cancer cells escaping from primary tumors can invade into the circulation as single CTCs or multicellular CTC clusters. Abbreviations: BM, basement membrane; CAF, cancer‐associated fibroblast; CTC, circulating tumor cell; EC, endothelial cell; ECM, extracellular matrix; EMT, epithelial‐to‐mesenchymal transition; MSC, mesenchymal stem cell; TAM, tumor‐associated macrophage

The EMT is the transition of epithelial cells to mesenchymal cells under certain physiological and pathological conditions, which was proposed by Greenberg and Hay in 1982.^28^, ^29^, ^30^ Studies in the last decades have shown that EMT is an important molecular event for epithelial tumor cells to gain invasiveness and plays an important role in the development and metastatic dissemination of malignant tumor cells.^31^ Tumor cells can obtain a higher mesenchymal phenotype through EMT, which manifests as reduced contact with surrounding cells and matrix, and enhanced cell migration and motility at the beginning of tumor cell invasion and metastasis.^30^ Therefore, interrupting or reversing EMT can inhibit the invasion of malignant tumor cells and reduce the rate of tumor metastasis.^32^ The process of EMT is shown in Figure 2.

Cancer cells undergoing and molecular EMT exhibit morphological changes, such as decreased expression of epithelial markers (e.g., E‐cadherin, zonula occludens‐1, and occluding) and increased expression of mesenchymal markers (e.g., N‐cadherin protein, fibroblast‐specific protein 1, and fibronectin).^33^, ^34^ Cell adhesion molecules, such as E‐cadherin, N‐ cadherin, and β‐catenin, are closely related to EMT and are regulated by EMT‐related transcription factors (EMT‐TFs), such as zinc finger E‐box‐binding homeobox 1/2 (ZEB1/2), snai1/2 and twist.^35^, ^36^, ^37^ The down‐regulation of E‐cadherin is accompanied by the up‐regulation of N‐cadherin, which reduces the adhesion of cancer cells to epithelial cells and increases their adhesion to stromal cells, leading to subsequent invasion of tumor cells into matrix.^38^ β‐catenin has also been found to promote tumor metastasis, mainly driven by the ectopic expression of β‐catenin.^39^ Vimentin has been found to promote cell invasion by regulating the E‐cadherin/β‐catenin complex.^40^ EMT‐TFs can be regulated by signaling pathways such as Wnt/β‐catenin, transforming growth factor‐beta (TGF‐β), and Notch; these factors work together to give tumor cells the characteristics required for metastasis under different conditions, thereby promoting the occurrence of metastasis.^41^, ^42^, ^43^, ^44^, ^45^

EMT is also involved in the process of tumor invasion and colonization in metastasis, which mainly depends on circulating tumor cells (CTCs) and CSCs.^41^, ^46^, ^47^ CTCs are divided into three groups, including epithelial CTCs, hybrid epithelial/mesenchymal phenotype CTCs, and mesenchymal CTCs.^48^ With the development of modern medical technology, tumor metastasis can be monitored throughout the process. Compared with epithelial CTCs, mesenchymal CTCs may be easier to metastasize because they are resistant to anoikis and chemotherapy and have a stronger ability to migrate to distant organs.^49^, ^50^ Previous studies reported that up‐regulating the expression of EMT‐TFs in breast cancer cells increased the expression of CSC‐specific cell markers, improved the ability to form spheroids, and accelerated tumor formation of breast cancer cells in mice.^51^ In non‐CSCs, the activation of EMT promotes their conversion to CSCs.^51^, ^52^, ^53^, ^54^ Tumor formation and metastasis rely on the tumor‐forming ability of tumor cells, which suggests that CSCs with tumor‐initiating ability are the crucial prerequisite for disseminated cancer cells to establish metastatic colonies.^55^

Another mechanism of EMT promoting metastasis is to protect tumor cells from immune cell‐mediated killing.^56^, ^57^, ^58^ Recent researches on breast cancer found that, compared with epithelial cancer cell lines, mesenchymal breast cancer cell lines can recruit more immunosuppressive regulatory T cells (Tregs) and M2 macrophages.^57^ Compared with epithelial cancer cells, mesenchymal cancer cells are less responsive to anti‐cytotoxic lymphocyte‐associated protein 4 (CTLA4) immunotherapy.^59^ In addition, the expressions of programmed death ligand‐1 (PD‐L1), PD‐L2, and B7 homolog 3 (B7‐H3) are up‐regulated in tumor cells that have undergone EMT, which can facilitate the immune escape of cancer cells.^58^, ^60^

During dormancy and colonization, the process of mesenchymal cells to transform to epithelial cells (MET) is required to help tumor cells survive in the foreign microenvironment.^31^ Indeed, the role of MET in promoting tumor cell colonization at a secondary site during metastatic growth has been confirmed in numerous studies.^31^, ^61^ It is worth noting that the tightly interconnected transfer network of EMT and MET has been proved to be subject to epigenetic regulation mediated by DNA methylation and histone modification.^62^, ^63^, ^64^ For example, in breast cancer, histone methyltransferase nuclear receptor‐binding SET domain 3 (NSD3) cooperates with enhancer of zeste homolog 2 (EZH2) and RNA polymerase II to stimulate the Notch pathway‐mediated E‐cadherin transcriptional inhibition to promote EMT and trigger tumor invasion and metastasis.^65^ During the initiation of EMT, histone demethylase lysine (K) ‐specific demethylase 6A (KDM6A) is inhibited, leading to the transcriptional inhibition of epithelial genes, whereas during the MET process, the expression of KDM6A is restored, thereby reactivating the epithelial gene by the trimethylation of histone 3 lysine 27 (H3K27me3) and reversing the phenotype.^66^, ^67^ Therefore, in general, a series of epigenetic regulation of tumor cells makes EMT dynamically change, giving tumor cells the ability to survive in different microenvironments.

### 2.2 The most important soil factor: TME

**FIGURE 3:**
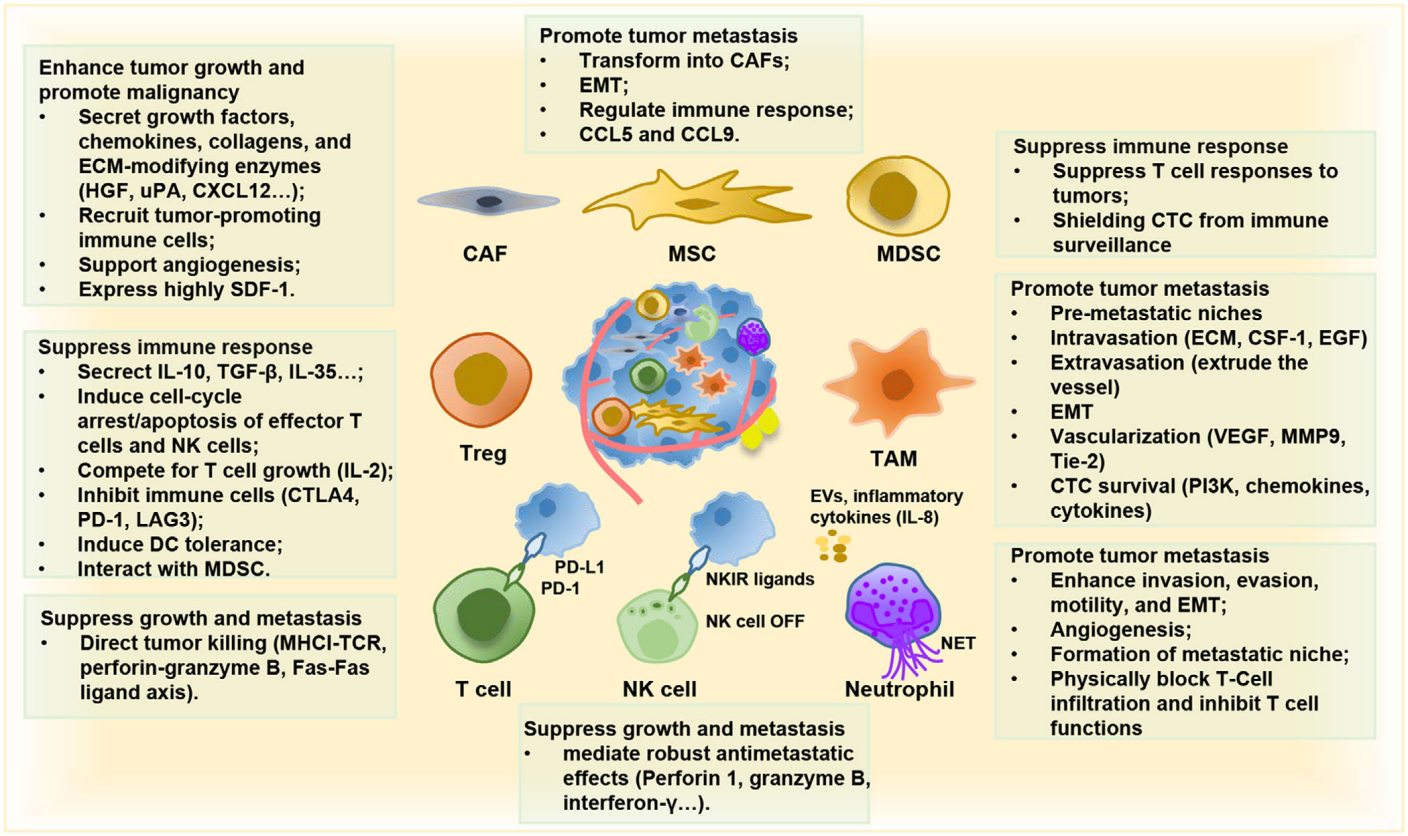
TME involved in the processes of invasion–metastasis cascade. The cellular components in TME can be classified into cancer cells, stromal cells, and immune cells. These cells interact with each other through ligand‐receptor interactions, and the secretion of cytokines, chemokines, exosomes, and extracellular vesicles, forming an evolving microenvironment. Cancer cells that are good at recruiting and establishing a supportive metastatic niche may be able to survive and initiate the process of proliferation and metastasis. The formation of the metastatic niche may occur before the arrival of cancer cells, also known as pre‐metastatic niches. Here, we summarized the role of important cellular components in TME in tumor metastasis

In addition to the characteristics of tumor cells, numerous studies have shown that TME is the soil for tumor cells and provides the necessary energy for tumor growth (Figure 3).^68^ In the past two decades, many achievements have been made regarding the impact of TME on cancer metastasis.^10^, ^11^, ^69^, ^70^ TME can promote the occurrence and development of tumor by affecting metabolism, secretion, immunity, structure, and function of tumor cells, thereby playing an essential role in the overall cascade of tumor metastasis.^12^, ^71^, ^72^ The members in TME are generally classified into two categories: cellular components and extracellular components. Cellular components mainly include immune cells, cancer‐associated fibroblasts (CAFs), mesenchymal stem cells (MSCs), and endothelial cells.^73^, ^74^, ^75^ Extracellular components, such as tumor‐secreted extracellular vesicles (EVs), growth factors, chemokines, and cytokines are also closely involved in tumor metastasis.^76^ Cellular components and extracellular components restrict and influence each other, which form a mutual feedback network.

#### 2.2.1 Immune cells

Under normal circumstances, the immune system will recognize foreign antigens to initiate autoimmunity and passive immunity to eliminate harmful pathogens. For this reason, tumor immunity has become a hot spot in the field of tumor research. Studies have pointed out that some types of immune cells are closely related to tumor occurrence and development and play an essential role in tumor metastasis.^72^, ^77^ Among the immune cells in TME, tumor‐associated macrophages (TAMs), Tregs, and neutrophils are noteworthy for their roles in tumor development and metastasis.

##### 2.2.1.1 TAMs

According to the expression of specific markers, differentiation status, and functional role in the immune system, macrophages are conventionally classified into two major phenotypes, M1 and M2, which can be converted to each other.^78^ M1 macrophages promote inflammation responses against tumor cells, whereas M2 macrophages tend to exert immune suppressive effects. M2 macrophages (M2‐TAMs) express abundant arginase‐1, mannose receptor, and scavenger receptors, and secrete a large number of anti‐inflammatory cytokines such as interleukin‐4 (IL‐4), interleukin‐10 (IL‐10), and interleukin‐13 (IL‐13).^79^, ^80^ In general, researchers tend to consider TAMs as M2‐like phenotype‐acquired macrophages.^81^ The cytokines and chemokines secreted by tumor cells or fibroblasts could recruit more M2‐TAMs in TME.^82^, ^83^, ^84^ TAMs are elevated in TME and are associated with poor clinical prognoses.^85^

The role of TAMs in promoting metastasis involves multiple mechanisms. First, TAMs participate in the regulation of the EMT process by enhancing the expression of N‐cadherin and snail, secreting various soluble factors such as IL‐1β, IL‐8, tumor necrosis factor‐α (TNF‐α), and TGF‐β, and secreting a number of proteolytic enzymes.^86^, ^87^, ^88^ Second, TAMs promote vascularization of tumor cells through stimulating the formation of new tumor vessels and the remodeling of the established vascular system into a more tortuous and leaky form.^89^, ^90^ Third, TAMs promote intravasation of tumor cell, favor tumor cell survival in the circulation, and promote extravasation of tumor cells.^91^ Last, TAMs are important determinants for the formation of pre‐metastatic niches. Notably, TAMs contribute to the immune escape of tumor cells throughout the metastasis process.^81^

##### 2.2.1.2 Tregs

There are mounting studies that convincingly demonstrated that Tregs play a prominent role in promoting metastasis. Tregs have a strong immunosuppressive function in TME by inhibiting adaptive and innate immune responses.^92^, ^93^, ^94^, ^95^, ^96^ Here, we summarized the main mechanisms.^97^ First, Tregs secrete inhibitory cytokines, including IL‐10, TGF‐β, and IL‐35.^98^, ^99^, ^100^ Tregs can inhibit the function of CD8+ T cells and dendritic cells (DCs) through membrane‐bound TGF‐β. For example, Tregs impede CD8+T cell‐mediated anti‐tumor immune responses by inhibiting interleukin‐2 (IL‐2) production and activating the TGF‐β signaling pathway.^92^ It has been reported that TGF‐β1 secreted by Tregs in breast cancer can induce the up‐regulation of IL‐17Rb and promote tumor lymph node metastasis.^101^ Second, Tregs can kill effector cells by granzymes and perforin and diminish the function of T cells and natural killer (NK) cells through inhibitory receptors, such as CTLA‐4, lymphocyte activation gene 3 (LAG‐3), and programmed cell death 1 (PD‐1).^102^, ^103^, ^104^ Tregs can also suppress CD8+ T cells and NK cells secretion of interferon‐γ (IFN‐γ).^105^ Olkhanud et al. found that chemokine receptor (CCR) 4+Tregs are mainly recruited at the lung metastasis site in breast cancer, and the Tregs secret β‐galactoside‐binding proteins to induce the apoptosis of NK cells, thereby promoting tumor metastasis.^95^ Third, Tregs affect effector cell functions by interfering with cell metabolism mainly through the following three ways: depriving IL‐2, promoting the production of adenosine, and transferring a large number of cyclic adenosine monophosphate to effector T cells.^106^, ^107^, ^108^, ^109^ Fourth, Treg induces DCs tolerance through the expression of inhibitory receptors CTLA‐4 and LAG‐3, with the latter further inhibiting T‐cell capacity through indoleamine 2,3‐dioxygenase.^110^, ^111^ Finally, factors produced by myeloid‐derived suppressor cells (MDSCs) and Tregs form positive feedback loops to reinforce the suppressive microenvironment.^112^, ^113^, ^114^

In addition, it has been found that Tregs could secrete vascular endothelial growth factor A (VEGF‐A) in metastatic ovarian cancer, thereby promoting angiogenesis and tumor cell dissemination.^115^ TGF‐β1 secreted by Tregs can promote tumor metastasis by enhancing EMT.^116^ Some preclinical studies have shown that Tregs were closely related to lymph node metastasis and peritoneal metastasis and suggested that Treg may be involved in pre‐metastasis niche reprogramming to promote metastasis.^101^, ^117^ Therefore, eliminating or alleviating the immunosuppressive state of Tregs may suppress the progression of tumor metastasis.

##### 2.2.1.3 Neutrophils

Neutrophils, as innate immune cells, protect the body from infection by foreign microorganisms and promote inflammatory responses under normal conditions.^118^ However, it is worth noting that neutrophils have been found to promote tumor metastasis in a variety of ways, including inhibiting anti‐tumor T cells and influencing tumor cell invasion.^119^ In a mouse model of sarcoma, pleomorphic neutrophils were found to reduce the expression of intercellular adhesion molecule 1 in tumor cells, thereby increasing tumor motility and facilitating the initiation of metastasis.^120^ In addition, pro‐metastatic neutrophils activate various signaling pathways to promote EMT by secreting pro‐EMT‐related cytokines, including IL‐8 and IL‐17A.^121^, ^122^ In the process of metastasis, neutrophils bind with CTCs in circulation to protect CTCs from immune killing, increase CTCs invasiveness, and promote metastasis.^123^ A large number of neutrophils were found in the secondary sites.^124^, ^125^ In terms of mechanism, recruited neutrophils promote the formation of pre‐metastatic niche and support angiogenesis mainly by forming an immunosuppressive microenvironment and secreting neutrophil elastase.^126^

#### 2.2.2 Cancer‐associated fibroblasts (CAFs)

CAFs are one of the most common fibroblasts in tumor tissues. CAFs are derived from a variety of different fibroblasts, including normal fibroblasts stimulated by exosomes, bone marrow MSCs induced by TGF‐β, and epithelial cells transformed by EMT.^127^, ^128^, ^129^ Since CAFs originate from many kinds of cells and have apparent heterogeneity, there is no clear marker that can distinguish CAFs from normal fibroblasts. CAFs are the primary source of various factors and enzymes of TME and are one of the critical members involved in tumor metastasis.

In previous studies, it was believed that the role of CAFs in tumor metastasis is mainly manifested in the reconstruction of ECM structure.^130^ However, current studies have found that CAFs promote EMT‐mediated tumor metastasis in ovarian cancer, bladder cancer, and breast cancer cells in various ways, including the secretion of TGF‐β and exosomes.^131^ The increased autocrine and paracrine TGF‐β signaling in the mesenchymal transcription factor forkhead box F2 (FOXF2) deficient base‐like breast cancer cells induces EMT to mediate tumor metastasis.^132^ In turn, TGF‐β silences FOXF2 expression by up‐regulating the post‐transcriptional regulator (miR‐182‐5p) of FOXF2 and promotes breast cancer metastasis.^133^ In addition, chemokine (C‐X‐C motif) ligand 11 (CXCL11) secreted by CAFs promotes the metastasis of liver cancer by up‐regulating the expression of circUBAP2.^134^ In order to gain a deeper understanding of the role of CAFs in the tumor, Wang et al. analyzed CAFs by single‐cell sequencing combined with RNA‐sequence and divided CAFs into four types.^136^ One of the subtypes has high glycolytic activity and is termed MeCAFs. It was found that pancreatic ductal adenocarcinoma patients with abundant MeCAFs had a higher risk of metastasis but had a significantly better response to immunotherapy.^136^ These results indicate that abnormal glycolysis directly or indirectly affects tumor metastasis. CAFs up‐regulate carnitine palmitoyltransferase 1A (CPT1A) to cause reduction of fatty acid oxidation, leading to peritoneal metastasis of colorectal cancer.^137^

Fibroblasts play an important role in both the primary tumor site and the secondary tumor site. Fibroblasts distributed in the metastatic site are called metastasis‐associated fibroblasts (MAFs), which promote angiogenesis and the formation of immunosuppressive microenvironment in pre‐metastatic niche.^138^ The main difference between MAFs and primary tumor‐related fibroblasts is that MAFs have a stronger ability to inhibit the anti‐tumor effect of immune cells, which is reflected explicitly in the higher levels of CC motif chemokines ligand 2 (CCL2), CXCL12, and interferon‐related genes secreted by MAFs.^139^ Additionally, in metastatic liver cancer and gastric cancer, it was found that the decrease of CD3+ infiltrating lymphocytes and the increase of TAM cells were related to the high levels of MAFs, partly due to the chemokine CXC receptor 4 (CXCR4) signal transduction in α‐smooth muscle actin (α‐SMA)+ MAFs.^140^ In another study conducted by Costa et al., fibroblasts that inhibit tumor immunity were discovered, named cancer‐associated fibroblasts subset 1 (CAF‐S1).^141^ Furthermore, it was found that the presence of CAF‐S1 in breast cancer promoted bone metastasis of cancer cells.^142^

#### 2.2.3 MSCs

More and more studies have recognized the importance of MSCs in regulating tumor metastasis at the initial tumor site and distant metastasis sites. At the beginning of tumor metastasis, MSCs support the generation of the cancer‐promoting microenvironment by transforming into CAFs and increase the motility of cancer cells through secreting growth factors and chemokines in autocrine and paracrine manners.^128^ Pendergast et al. co‐cultured MSCs and lung cancer cells and found that MSCs can mediate EMT to promote lung cancer metastasis by activating the abelson‐ matrix metalloprotein 9 (ABL‐MMP9) signaling pathway.^143^ In the late stage of tumor metastasis, MSCs participate in the colonization and metastatic growth of tumor cell. For example, in ovarian cancer, MET of cancer‐associated MSCs mediated by Wilms’ tumor 1 (WT1) and EZH2 promote metastatic tumors growth in distant organs.^135^

MSCs can also promote tumor metastasis by regulating anti‐tumor immunity. As an immune checkpoint, PD‐L1 is one of the crucial members involved in tumor immune escape and treatment resistance.^144^ Studies have found that PD‐L1 is highly expressed on tumor‐associated MSCs, and MSCs can up‐regulate the expression of PD‐L1 in a variety of tumor cells. Hamidreza et al. reported that MSCs promoted the up‐regulation of PD‐L1 expression in breast cancer cells by secreting CCL5.^145^ Sun et al. found that IL‐8 secreted by MSCs can increase the expression of PD‐L1 in gastric cancer cells.^146^ Further experiments suggested that MSCs can increase the binding of PD‐L1 to the transcription factor CCCTC‐binding factor, thereby affecting the stemness of gastric cancer cells and leading to tumorigenesis.^147^

#### 2.2.4 Tumor‐associated endothelial cells (TECs)

The role of TECs in metastasis is complex. In the process of tumor metastasis, TECs play an important role in obtaining anti‐anoikis properties of CTCs, which is one of the mechanisms by which TECs promote tumor cell metastasis to secondary organs.^148^ In addition, studies have found that biglycan in TECs is significantly higher than that in normal endothelial cells.^149^ TECs‐biglycan activates nuclear factor‐κB (NF‐kB) and extracellular signal‐regulated kinase (ERK) signal transduction to stimulate the metastasis of tumor cells expressing toll‐like receptors (TLR) and promote tumor angiogenesis of tumors by activating the TLR signaling pathway.^150^ IL‐6 secreted by tumor cells can activate the signal transducers and activators of transcription 3 (STAT3)‐VEGF pathway of lymphatic endothelial cells and encourage lymphatic metastasis of the tumor.^151^

#### 2.2.5 Extracellular matrix (ECM)

ECM is a network of various proteins represented by collagen and macromolecules, which maintain tissue structure outside tumor cells.^18^ A hallmark of metastasis is ECM degradation.^152^ In the process of metastasis, it is necessary to enhance the invasion ability of tumor cells and reconstruct the ECM structure.^153^ ECM reconstruction is a relatively complex process, which requires the participation of multiple chemokines.^154^, ^155^ Matrix metalloproteinases (MMPs) are a group of enzymes that can directly degrade collagen and multiple connexins in the process of ECM degradation. Among matrix MMPs family, MMP‐2 and MMP‐9 are the two important enzymes involved in the EMT process, which specifically degrade gelatin, collagen, elastin, and fibronectin, thereby inducing invasion and metastasis of tumor cells.^156^ Furthermore, Musashi‐1 can promote the degradation of ECM by upregulating Timp3 to encourage the expression of MMP‐9 in breast cancer, and thus regulating cell‐ECM adhesion.^157^

#### 2.2.6 Cytokines and chemokines

The involvement of various cytokines/chemokines, along with their receptors and signaling axis in the promotion of tumor metastasis has been well‐studied. The convoluted cross‐talk between various cell types and these secreted factors helps drive the sequence of events that lead to tumor metastasis.^158^, ^159^ These cytokines/chemokines are involved in ECM remodeling, tumor invasion, EMT, angiogenesis, pre‐metastatic niche reprogramming, extravasation, and modulating stromal cells and immune cells.^115^, ^154^, ^160^, ^161^, ^162^ The cytokines/chemokines including TNF‐α, IL‐8, CCL20, CXCL5, CXCL12.^163^, ^164^, ^165^, ^166^ CXCR2, CXCR3, and CXCR4, up‐regulate EMT‐TFs (snail and ZEB1), promote MMP‐2 expression, and accelerate EMT of cancer cells, thereby promoting tumor metastasis.^160^, ^165^, ^166^ Cytokine IL‐8 mainly down‐regulates E‐cadherin through activating the Wnt/β‐catenin pathway in ovarian cancer, thereby promoting tumor invasion.^163^ Some cytokines can promote the expression of chemokines and mesenchymal transformation. For example, TNF‐α has been reported to promote the up‐regulation of CXCL10 and activate the phosphoinositide3‐kinase/protein kinase B (PI3K/AKT) pathway to inhibit the phosphorylation of GSK‐3β, leading to the up‐regulation of snail and promoting tumor metastasis.^160^

Cytokines/chemokines, such as VEGF, TGF‐β, IL‐1, CXCL8, and CCL21 are involved in promoting angiogenesis and lymphatic formation.^167^, ^168^, ^169^, ^170^, ^171^ Among the known pro‐angiogenic factors, VEGF is the most effective cytokine in promoting angiogenesis. By directly binding to receptors on endothelial cells, VEGF induces endothelial cell proliferation and promotes tumor metastasis.^167^ VEGF‐C and VEGF‐D in the VEGF family can activate the generation of lymphatic vessels, recruit chemokines, such as CCL21, CCL27, and CCL28, to lymphatic vessels and promote the occurrence of lymph node metastasis.^172^, ^173^ Cytokines/chemokines also play an indispensable role in promoting the recruitment of immune cells in the tumor microenvironment. For example, the recruitment of Treg requires the participation of CCL20 and CCL22, while the recruitment of TAMs requires the participation of CCL2.^174^, ^175^, ^176^, ^177^ Single‐cell sequencing analysis revealed that chemokine CCL5 is an important mediator of CTC immune escape.^162^ CCL5 can promote immune escape and metastasis of CTCs by recruiting Tregs, which further suggests that chemokines can promote immune escape.^162^ In addition, MDSCs secrete TGF‐β in esophageal cancer, which can increase the expression of PD‐1 on tumor‐infiltrating CD8+ T cells, leading to immunotherapy resistance.^178^, ^179^

Cytokines and chemokines are involved in the organotropism of tumor metastasis. Lung metastasis is associated with the high levels of CXCL1, CXCL9, and CXCL10 secreted by human pulmonary artery endothelial cells or lung fibroblasts.^179^, ^180^ Ricardo et. Al. reported that blocking CXCL5/CXCR2 signaling can inhibit the bone colonization of breast cancer cells.^181^ Blocking CXCL12/CXCR4 signaling significantly impair the metastasis of breast cancer cells to regional lymph nodes and lung, which indicate that chemokines and their receptors play a key role in determining the destination of metastasis.^182^, ^183^ Overall, the potential of cytokines and chemokines to induce metastasis is mainly achieved by promoting EMT, angiogenesis, immunosuppression, and pre‐metastatic niche reprogramming.

#### 2.2.7 Tumor‐secreted extracellular vesicles

EVs secreted by tumors are the key mediators of cell‐to‐cell contact in the local and remote microenvironments.^184^ EVs are exceptional cargo for various nucleic acid, proteins, and lipids in TME, and play a prominent role in the metastasis of the tumor to distant organs.^183^, ^184^, ^185^, ^186^, ^187^, ^188^, ^189^, ^190^ Tumor‐associated EVs in breast cancer can promote breast cancer colonization by changing the composition and structure of lung tissue fibroblasts.^185^ It was found that EVs secreted by bladder cancer cells mediate their intercellular communication with human lymphatic endothelial cells through long non‐coding RNA (lncRNA) ELNAT1 and promote lymph node metastasis in a SUMOylation‐dependent manner.^186^

In nasopharyngeal carcinoma (NPC), EVs migrate from highly metastatic to poorly metastatic NPC cells, mediate intercellular communication, and enhance the metastatic potential of poorly metastatic NPC cells by inducing the up‐regulation of epidermal growth factor receptor (EGFR) and down‐regulation of reactive oxygen species (ROS).^191^ Mechanistically, overexpression of EGFR mediated by EGFR‐rich EVs down‐regulates intracellular ROS levels through regulating PI3K/AKT pathway, thereby promoting the metastatic potential of NPC with poorly metastatic ability.^191^

The role of EVs in tumor metastasis depends on their components; the function of EVs may be different for different tumor metastasis sites. For example, in brain metastasis, EVs mainly mediate pre‐metastasis regulation by destroying the blood‐brain barrier or by distinct increasing the expression of cell migration‐inducing and hyaluronan‐binding protein in brain‐tropic EVs.^187^, ^188^ In hepatocellular carcinoma, nidogen 1 (NID1) in EVs promote tumor cell colonization and extrahepatic metastasis by enhancing angiogenesis and lung endothelial permeability and promoting the formation of pre‐metastasis niches in the lung.^189^ EVs‐NID1 also activates fibroblasts that secrete TNF receptor 1 (TNFR1), promote lung colonization of tumor cells, and augment the growth and motility of cancer cells.^189^ EVs in bone metastasis mainly mediate cancer‐induced osteolysis to produce a suitable microenvironment for tumor cells.^190^ In addition, EVs can also promote the formation of pre‐metastasis niches by stimulating CAFs, inducing MSC differentiation, and regulating tumor immunity.^183^

#### 2.2.8 Neutrophil extracellular traps (NETs)

NETs are net‐like structures composed of DNA histone complexes, and proteins released by neutrophils in response to infection or inflammatory cytokines were discovered by Brinkmann et al. in 2004.^192^ NETs are initially discovered to be one of the host defense mechanisms of neutrophils against pathogens, and they are also involved in the progression of sterile inflammation‐related diseases, such as autoimmune diseases, diabetes, and carcinoma.^193^ In the absence of infection, NETs can also be stimulated by cancer cells and CAFs, and hijack anti‐tumor immune system to promote tumor cell proliferation and metastasis.^193^

The levels of plasma NETs in patients with lung cancer, cervical cancer, and pancreatic cancer are significantly higher than those in healthy controls and are related to tumor recurrence and metastasis.^194^ In tumor metastasis, NETs are mainly involved in the spread of cancer cells and awakening dormant tumor cells. NETs can induce EMT of primary tumor cells in the early stage of metastasis and lead to the activation of inflammatory signaling pathways such as NF‐kB and STAT3, thereby enhancing the mobility and invasiveness of cancer cells and promoting metastatic growth.^195^, ^196^

In the circulation, NETs wrap CTCs to avoid contact between CTCs and immune cells and can also form a physical barrier for immune escape.^197^, ^198^ The molecular mechanisms by which NETs can trap CTCs have been investigated, and it has been found that expression of β1‐integrin on both cancer cells and NETs is important for the adhesion of CTCs to NETs.^197^, ^198^ After spreading to other sites, cancer cells from primary tumors often do not start growing immediately but enter a dormant state. Disseminated cancer cells can remain dormant for years or even decades before they relapse or “wake up” as metastatic cancer.^199^ Studies have found that the formation of NETs induced by sustained inflammation is required for awakening dormant cancer.^199^ Two NET‐associated proteases (neutrophil elastase and MMP9) sequentially cleave laminin, and then NET‐remodeled laminin facilitates the proliferation of dormant cancer cells by activating integrin α3β1 signaling and focaladhesion kinase (FAK)/ERK/myosin light chain kinase (MLCK)/Yes associated protein (YAP) signaling.^199^

### 2.3 A double‐edged knife in tumor metastasis: Autophagy

Autophagy is generally defined as a lysosome‐dependent mechanism of intracellular degradation and recycling process, which is highly conserved in all eukaryotes.^200^, ^201^, ^202^ Several forms of autophagy have been discovered. In all types of autophagy, autophagosomes are the hallmark morphological characteristics, and the formation of autophagosomes is the core process of this dynamic process.^200^, ^203^ Autophagy is a dynamic physiological process that maintains cell homeostasis when the body encounters stress, including nutrient deprivation, hypoxia, and infection.^204^, ^205^ It has been reported that autophagy plays a key role in the prevention of cancer and other diseases by promoting cellular senescence and antigen presentation and preventing genomic instability and necrosis.^206^ However, autophagy also plays a vital role in the occurrence and progression of tumor.^207^, ^208^ In many types of tumors, including melanoma, breast cancer, and liver cancer, the expressions of autophagy‐related proteins light chain 3 and beclin‐1 in tumor tissues of patients with distant metastasis are higher than in tumor tissues of patients with non‐metastatic tumor, suggesting that autophagy is closely related to tumor metastasis.^209^, ^210^, ^211^ Partly due to the heterogeneity of tumor cells, autophagy is a double‐edged sword in the process of tumor metastasis.^212^

In the early stage of metastasis, autophagy mainly suppresses the occurrence of metastasis by regulating the anti‐tumor immune response.^213^, ^214^, ^215^ Autophagy‐related protein 5(ATG5) is considered to be closely related to autophagy. In pancreatic ductal adenocarcinoma, mice with ATG5 knockout developed more tumors and metastases than control mice. Moreover, compared with the control mice, the expression of cytokines that regulate macrophage chemoattraction and differentiation into M2 macrophages was up‐regulated in the tumors of mice with ATG5 knockout. Meanwhile, the number and activity of M2‐TAMs were substantially higher in ATG5 knockout mice, indicating that autophagy can inhibit TAMs infiltration and limit tumor metastasis.^214^ In addition, studies have found that autophagy can stimulate tumor‐related spontaneous inflammation by releasing high mobility group box 1 and enhance the antitumor immune response of DCs to limit metastasis.^215^ In the later stage of tumor metastasis, autophagy reduces the adhesion between tumor cells and ECM by regulating the activity of the Rho family so as to promote tumor migration and invasion.^216^ In addition, autophagy can enhance tumor invasion and promote the progress of tumor metastasis by inhibiting anoikis of CTCs, maintaining the stemness of CSCs, and reawakening dormant tumor cells.^210^, ^217^, ^218^

Another significant aspect of autophagy in metastasis is involved in the regulation of EMT. P53, an oncogene, also plays an important role in regulating the relationship between EMT and autophagy.^219^, ^220^ Under normal circumstances, P53 exists in the cytoplasm and inhibits autophagy. However, under cellular stress, P53 translocates from the cytoplasm to the nucleus and induces autophagy.^221^, ^222^ In addition to autophagy, recent studies have found that P53 inhibits EMT and metastasis by affecting the TFs involved in the EMT process.^223^

It is well known that AKT/ mammalian target of rapamycin (mTOR) signaling pathway participates in modulating the EMT process.^224^ A large number of studies have found that oncogenes activate mTOR‐related pathways to promote tumor metastasis by inhibiting autophagy.^225^ Suppressor of cytokine signaling‐5 (SOCS5), a member of the SOCS protein family, has also been found to promote tumor cell invasion and metastasis in hepatocellular tumors by up‐regulating PI3K/Akt/mTOR‐mediated autophagy pathway.^226^ Interestingly, opposite findings have been reported in NPC. Annexin A1 (ANXA1) inhibits autophagy and promotes EMT by activating PI3K/Akt/mTOR pathway.^227^ ANXA1 inhibits autophagy by activating PI3K/Akt/mTOR pathway, leading to the up‐regulation of tumor cell migration and invasion capabilities, EMT‐like changes, and metastasis in vivo.^227^ During this process, autophagy inhibited by ANXA1 induces EMT‐like alterations, possibly by inhibiting autophagy‐mediated Snail degradation.^227^

Autophagy can also be induced by activating the adenosine monophosphate activated protein kinase (AMPK) pathway that promotes tumor metastasis.^228^ For example, tight junction protein 1 (CLDN1) promotes EMT and metastasis of esophageal cancer cells by triggering autophagy in vivo and in vitro.^229^ Mechanically, CLDN1 induces autophagy by up‐regulating the expression of Unc‐51‐like autophagy activating kinase 1 (ULK1) through AMPK/signal transducer and activator of transcription 1 (STAT1)/ULK1 signaling pathway.^229^, ^230^ In general, autophagy is a double‐edged sword in the process of tumor metastasis, and the mechanisms involved are very intricate and require further research.

### 2.4 Lipid metabolism

Under hypoxic conditions, tumor cells mainly rely on glycolysis to obtain sufficient energy.^231^ This metabolic mode different from normal cells is also called tumor‐related metabolic rearrangement.^232^ Glycolysis is essential to maintain the malignant proliferation behavior of the tumor, but there are relatively few studies on the effect of lipid metabolism on tumor progression. Obesity has been recognized as a risk factor for cancer, so investigating lipid metabolism in the tumor is necessary.^233^, ^234^ The results of researches in the past 10 years have shown that abnormal lipid metabolism in the TME is one of the key steps involved in tumor metastasis.^235^ Therefore, correcting abnormal lipid metabolism may prevent the occurrence of tumor metastasis.

It has been found that a variety of enzymes involved in lipid anabolism and catabolism are related to tumor metastasis, including adenosine triphosphate (ATP) citrate lyase (ACLY), fatty‐acid synthase (FASN), stearoyl‐CoA desaturases (SCD), and monoacylglycerol lipases.^236^ ACLY and SCD‐1 are associated with facilitated colon cancer metastasis, and FASN can promote retroperitoneal metastasis of ovarian cancer.^237^, ^238^ Identifying tumor metastasis initiating cells is considered to be one of the most challenging problems in the field of tumor metastasis research. In recent years, fatty acid receptor CD36 has been shown to play an essential role in the metastasis of oral cancer because CD36 is positively correlated with lymph node metastasis and can be used as a marker of metastasis initiating cells.^239^ Palmitic acid or a high‐fat diet can enhance the metastatic ability of CD36+ tumor cells. Subsequent studies have found that CD36+ cells are associated with a poor prognosis of ovarian cancer, and blocking CD36 can attenuate tumor metastasis.^240^ These findings suggest that the initiating cells of tumor metastasis may depend on lipids, and blocking CD36 can reverse tumor metastasis.

With the development of proteomics and metabolomics, new discoveries have been made about cancer metabolic disorder and its correlation with metastasis. In breast cancer, it was found that the levels of phospholipid are different between mammary epithelial cells and breast cancer cells, as well as between breast cancer cells with different levels of aggressiveness.^241^ In short, lipid metabolic reprogramming is associated with breast cancer carcinogenesis and metastasis.^241^ In pancreatic cancer, fatty acid synthesis was found to maintain the stemness of pancreatic cancer cells, indicating that abnormal lipid metabolism can make cancer cells more invasive and promote the spread of tumor cells.^242^ Promoting cholesterol biosynthesis may promote tumor metastasis.

More and more molecules regulating lipid metabolism have been proved to promote tumor metastasis by regulating EMT, including apolipoprotein C, sterol regulatory element‐binding transcription protein 1, stromal‐interaction molecule 1, human hydroxysteroid dehydrogenase‐like 2, and cytosolic phospholipase A2α.^243^, ^244^, ^245^, ^246^ In triple‐negative breast cancer, nicotinamide adenine dinucleotide phosphate (NADP) steroid dehydrogenase‐like (NSDHL), a cholesterol metabolic enzyme, has been reported to be a potential metastatic driver.^247^ The functions of NSDHL rely on its enzyme activity in the biosynthesis of cholesterol and is mediated by the NSDHL‐TGFβR2 signaling pathway.^247^

TME has a unique lipid structure, which is characterized by being rich in sphingolipids and cholesterol and is called lipid rafts.^248^ The role of lipid rafts in tumor metastasis is different from other lipid metabolism‐related regulatory molecules. Rina et al. reported that palmitoylated CD44 is wrapped in lipid rafts, and its binding to pro‐migration binding partners (such as Ezlin) is restricted, thereby inhibiting cancer metastasis and spread.^249^ Moreover, another study reported that squalene synthase could promote lung cancer metastasis by activating TNFR1, NF‐Κb, and matrix metallopeptidase 1, and destroying the lipid raft structure.^250^ Therefore, lipid rafts mainly play an inhibitory role in tumorigenesis and metastasis. For cells in TME, abnormal lipid metabolism may be a potential mechanism to promote tumor metastasis. For example, lipid accumulation in TME can lead to CD8+T cell dysfunction and promote TAMs differentiation.^251^, ^252^ In addition, the abnormal elevation of fatty acid synthase in CAFs may enhance the aggressiveness of tumor cells.^253^ Therefore, lipid metabolism reprogramming plays an important role in regulating the formation of the pro‐metastatic TME.

### 2.5 Long non‐coding RNA

LncRNA is a standard non‐coding RNA with a length of more than 200.^254^ Researches in recent years have shown that lncRNA plays an important role in tumorigenesis.^254^ In fact, lncRNA plays a double‐edged role in tumor metastasis. LncRNA may be involved in all the processes of metastasis, including EMT, tumor invasion and migration, and tumor cell colonization in secondary sites. We briefly summarized the representative lncRNA that have been shown to be relevant with metastasis in vivo and in vitro (Table 1).^255^, ^256^, ^257^, ^258^, ^259^, ^260^, ^261^, ^262^, ^263^, ^264^, ^265^, ^266^, ^267^, ^268^, ^269^, ^270^, ^271^, ^272^, ^273^, ^274^, ^275^, ^276^, ^277^, ^278^, ^279^, ^280^

**TABLE 1:** Important metastasis‐related long non‐coding RNAs

**Metastasis‐related long non‐coding RNA**	**Cancer type**	**Function**	**Effect**	**Reference**
H19	Bladder cancer	Bind to enhancer of zeste homolog 2 (EZH2) to downregulate E‐cadherin	Inhibit	^255^
	Prostate cancer	Encode miR‐675 to mediate the down‐regulation of TGF‐β1	Promote	^258^
	Colorectal cancer	Upregulate zinc finger E‐box‐binding homeobox 1/2 protein	Promote	^257^
PNUTS	Breast cancer	Competitive sponge for miR‐205 and miR‐200 and enhancing epithelial‐mesenchymal transition (EMT)	Promote	^259^
LINC00460	Colon cancer	Enhance the expression of high‐mobility group AT‐hook 1	Promote	^260^
Lnc01232	Pancreatic cancer	Upregulate HNRNPA2B1, and activate the MAPK/ERK signaling	Promote	^261^
MALAT1	Colorectal cancer	Regulate the miR‐106b‐5p via SLAIN2	Promote	^262^
TPA	Breast Cancer	Activate TGF‐β signaling pathway	Promote	^263^
PVT1	Colon Cancer	Downregulate tumor suppressor miR‐152‐3p	Promote	^264^
LIMT	Breast cancer	Suppress tumor cells motility	Inhibit	^265^
SPRY4‐IT1	Bladder cancer	Bind to miR‐101‐3p to upregulate the expression of EZH2	Promote	^266^
TRERNA1	Gastric cancer	Regulating CDH1 to upregulate SNAI1	Promote	^267^
NEF	Hepatocellular carcinoma	Suppress Wnt/β‐catenin signaling to activate expression of FOXA2	Inhibit	^268^
HOXD‐AS1	Hepatocellular carcinoma	Competitive bind to miR‐130a‐3p to upregulate the expression of EZH2 and MMP2	Promote	^269^
CYTOR	Colon cancer	Activate Wnt/β‐catenin signaling to enhance EMT	Promote	^270^
JPX	Lung cancer	Activate Wnt/β‐catenin signaling pathway to upregulate Twist1 expression	Promote	^271^
LINC00662	Colon cancer	Activate extracellular signal‐regulated kinase (ERK) signaling pathway	Promote	^272^
ID2‐AS1	Hepatocellular carcinoma	Activate HDAC8/ID2 signaling pathway to decrease Twist expression	Inhibit	^273^
RPPH1	Colorectal cancer	Interact with TUBB3 to reduce E‐cadherin levels and induce macrophages M2 polarization	Promote	^274^
SATB2‐AS1	Colorectal cancer	Regulate SATB2 to decrease MMP9 and vimentin	Inhibit	^275^
NORAD	Lung cancer Breast cancer	Bind and sequester S100P to suppress S100P pro‐metastatic signaling pathway	Inhibit	^276^
URRCC	Renal cancer	Enhance EGFL7 expression to suppress P‐AKT/FOXO3 signaling pathway	Promote	^277^
FEZF1‐AS1	Colorectal cancer	Activate PKM2/signal transducers and activators of transcription 3 signaling pathway	Promote	^278^
ADAMTS9‐AS2	Salivary adenoid cystic carcinoma	Activate PI3K/Akt and MEK/ERK signaling pathway	Promote	^279^
GAS5	Pancreatic cancer.	Regulate miR‐221/SOCS3 to suppress EMT and cancer stem cells self‐renewal	Inhibit	^280^

As mentioned above, EMT is an essential step in the initiation of tumor metastasis. It has been reported that LncRNA H19 can mediate the transformation of tumor cells into mesenchyme and is significantly elevated in bladder cancer, breast cancer, and colorectal cancer.^255^, ^256^, ^257^ H19 directly binds to EZH2 in bladder cancer, resulting in a reduction in EMT epithelial marker E‐cadherin.^255^ In colorectal cancer, H19 acts as a competing endogenous RNA, leading to the up‐regulation of ZEB1/2 protein expression.^257^ However, in prostate cancer, the opposite phenomenon has been observed, that is, H19 inhibits tumor metastasis, mainly by encoding miR‐675 to mediate the down‐regulation of TGFBI.^258^ Similar to H19, lncRNA‐PNUTS serves as a competitive sponge for miR‐205 and miR‐200, leading to the up‐regulation of EMT in breast cancer.^259^ LncRNA LINC00460 is increased in colon cancer, and it induces EMT and promoteS tumor growth and metastasis by enhancing the expression of high‐mobility group AT‐hook 1 and decreasing the expression of E‐cadherin.^260^

The influence of lncRNA on tumor invasion and migration is also one of the mechanisms by which it affects tumor metastasis. For example, colon cancer‐associated transcript 2 (CCAT2) and RNA associated with metastasis‐11 (RAMS11) have been reported to participate in the process of tumor invasion and migration. CCAT2 is related to the stability of microsatellites; knockdown of CCAT2 inhibits tumor invasion and migration in mouse models. CCAT2 may interact with TCF7L2 to activate Myc transcription and Wnt signaling pathways and thus promote metastasis.^281^, ^282^ RAMS11 is overexpressed in colon cancer with liver metastasis. Knockout of RAMS11 gene using CRISPR‐Cas9 technology can reduce the invasion and migration of colon cancer cells in vitro and reduce liver metastasis in mouse model.^283^ Lnc01232 was found to promote metastasis by inhibiting ubiquitination, upregulating HNRNPA2B1, and activating the mitogen‐activated protein kinase/extracellular signal‐regulated kinase (MAPK/ERK) signaling pathway in pancreatic cancer.^261^

As the first lncRNA found to be associated with metastasis, metastasis‐associated lung adenocarcinoma transcript 1 (MALAT1) has been shown to be associated with poor prognosis of lung cancer and breast cancer, and its expression is significantly increased in patients with metastatic cancer.^284^, ^285^ In the breast cancer mice model lacking the promoter of MALAT1 or MALAT1, tumor differentiation and E‐cadherin are increased, whereas lung metastasis is significantly reduced, indicating that MALAT1 may serve as essential factors for tumor cell colonization at distant metastasis sites.^285^

With the progress in understanding the functions of lncRNAs, increasing evidence has indicated that lncRNAs play a role in the physiological and pathological processes of malignancies. Although many findings need further verification, it is certain that lncRNAs can carry out diverse functions in carcinogenesis and metastasis. However, the specific role and mechanisms of different lncRNAs in cancer metastasis need to be further elucidated.

## 3 INTERVENTIONS FOR TUMOR METASTASIS

Metastatic disease is the major contributor to cancer‐related mortality. Therefore, there is an urgent need for effective cancer treatments that can eliminate large solid tumors and disseminated and metastatic nodules, while simultaneously preventing tumor recurrence. With the deepening understanding of the molecular mechanism of tumorigenesis and metastasis in recent years, a plethora of treatment approaches, including targeted therapy and immunotherapy have been established for antitumor treatments.^13^, ^286^, ^287^ Although the emergence of new therapies, such as those based on immune checkpoint inhibitors (ICIs), is rapidly changing the treatment modality of metastatic patients in some cases, the current standard of cancer treatment for localized disease is still usually based on surgery, chemotherapy, and radiotherapy.^288^, ^289^ In addition, the combination therapies are receiving more attention and are being actively evaluated.^290^ We summarize the interventions for metastatic diseases based on preclinical and clinical evidence.

### 3.1 Surgery, chemotherapy, and radiotherapy

Surgery, chemotherapy, and radiotherapy are the three cornerstones of tumor treatment. According to the purpose of treatment, surgical treatment can be divided into radical surgery, local surgery, and palliative surgery.^291^ For patients with early‐stage tumor, radical surgery can prevent and reduce tumor recurrence and metastasis. However, for most patients, due to the insidious tumor‐related symptoms, distant metastasis has already occurred at the time of diagnosis. In the past, it was considered that metastatic tumors cannot be treated surgically, but a number of recent studies and clinical practices have confirmed that local surgery can be performed on some patients with localized metastasis, which can reduce the tumor burden and may achieve the goal of radical cure.^292^, ^293^, ^294^, ^295^ For example, palliative surgery for patients with intraperitoneal metastases such as liver cancer and ovarian cancer can alleviate their symptoms and improve their quality of life.^296^, ^297^

For intervention and treatment of metastatic tumors, surgery alone is not enough. Dormant CTCs is the main cause of early postoperative metastasis. Therefore, many clinical studies have been carried out to reduce the probability of tumor metastasis. Neoadjuvant chemotherapy and adjuvant chemotherapy are currently widely used in the treatment of cancer because these interventions can kill CTCs and reduce the possibility of metastasis while reducing the size of the preoperative tumor and preserving some functional organs.^298^, ^299^, ^300^, ^301^ Except for a small number of patients, chemotherapy, such as 5‐FU, Adriamycin, and platinum, is the first‐line choice for most patients with advanced cancer.^301^, ^302^, ^303^, ^304^ It has been observed that diverse malignancy patients with metastatic lesions benefit from chemotherapy, especially patients with lymphoma and leukemia, and some patients can even obtain a durable response.^305^, ^306^

However, conventional maximum‐dose chemotherapy cannot be tolerated by many patients with advanced cancer due to severe side effects and ultimately leads to treatment failure. In addition, it has been found that chemotherapy may induce the production of pro‐metastatic cytokines and chemokines, thereby inducing tumor metastasis.^307^ Therefore, many studies have been carried out to optimize chemotherapy regimens, and some progress has been made. Recent studies have shown that metronomic chemotherapy can inhibit angiogenesis and reduce the expression of pro‐metastatic cytokines and chemotaxis, thereby reducing tumor recurrence and the distant spread of tumor cells.^308^, ^309^ These results indicate that low‐dose metronomic chemotherapy may be effective in the prevention and treatment of tumor metastasis.

Radiotherapy has been recognized as a radical treatment for some early‐stage cancer, such as NPC, and progress has also been made in the treatment of patients with metastatic cancer.^295^, ^310^ Compared with radiotherapy alone, the combination of radiotherapy and other therapies has better therapeutic effects on metastatic cancer, especially when combined with chemotherapy. For locally advanced NPC, concurrent chemoradiotherapy has been approved as a standard regimen by the National Comprehensive Cancer Network Guidelines.^310^ Recently, two Phase III clinical trials from China proposed that induction chemotherapy before concurrent chemoradiotherapy can significantly improve the prognosis of patients with NPC.^311^, ^312^ It can be reflected that multidisciplinary combination therapy will be the main means to improve the prognosis of patients with metastatic cancer.

### 3.2 Targeted therapy for metastasis

For most patients with advanced‐stage cancer, surgery and radiotherapy are difficult to achieve satisfactory therapeutic effects, which is also the reason for the high tumor‐related mortality. In the process of tumor metastasis from initiation to colonization, the occurrence of each step is the result of the joint action of some specific genes and signaling pathways. Blocking one of these steps may block the formation of metastases. Therefore, the development of drugs targeting these targets may provide an alternative for patients with advanced tumors.

#### 3.2.1 Targeting EMT and cell motility

Blocking EMT is one of the key strategies to prevent tumor cells from spreading from the primary tumor site. As a marker of mesenchymal cells, N‐cadherin is elevated in metastatic tumors and is associated with a poor prognosis.^33^, ^38^ ADH‐1 is the first humanized antibody selectively targeting N‐cadherin, which has been proved to improve the prognosis of patients with tumor‐expressing N‐cadherin.^313^ Recently, a study showed that blocking N‐cadherin by ADH‐1 can also inhibit the expression of PD‐L1 and the recruitment of Tregs in TME and augment the cytotoxicity of tumor‐infiltrating lymphocytes (TILs) against tumor cells.^314^ The steroid receptor coactivator (SRC) tyrosine kinase family is one of the central members that mediate EMT.^45^ Drugs targeting SRC, including dasatinib, bosutinib, and saracatinib, have been proved to inhibit tumor growth and prolong patient survival.^315^ When combined with other treatment modalities, the anti‐tumor effect is stronger. In a Phase III prospective clinical trial (NCT01584648), the 3‐year progression‐free survival rate of patients receiving dabrafenib plus trametinib was 22% and 12% for patients receiving dabrafenib monotherapy, and the 3‐year overall survival rate was 44% and 32%, respectively. Furthermore, in the subgroup with normal lactate dehydrogenase and less than three metastatic sites, the 3‐year overall survival rate of patients receiving combination therapy reached 62%.^316^

The motility of tumor cells is one of the critical factors that determine whether the metastasis can proceed smoothly, and the integrin family can affect cell motility by regulating cell adhesion.^37^ Therefore, targeting the integrin family can weaken the motility of tumor cells. Currently, drugs targeting the integrin family mainly include cilengitide, intetumumab, 264RAD, and MK‐0429, which have been proven to prolong the survival of cancer patients in preclinical studies and prospective clinical studies.^317^, ^318^, ^319^, ^320^ The allosterisms of actin and myosin in ECM promote structural remodeling and tumor cell polarization during tumor cell migration; Ras homolog gene family, member A (RhoA), MMP, and non‐muscle Myosin‐II are involved in the allosterisms of these two proteins.^130^ Small molecule inhibitors of molecules regulating actin and myosin have been shown to attenuate tumor cell motility and inhibit tumor metastasis in vivo and in vitro, but their effectiveness in humans needs to be confirmed by further clinical studies.^156^, ^321^

After separation from ECM, only a small percentage of cells can survive and achieve subsequent metastasis. The main reason is that this small part of tumor cells has acquired the properties of anti‐anoikis.^19^, ^322^ Therefore, tumor metastasis can be further inhibited by promoting tumor cell anoikis and eliminating anoikis resistance. T0070907 inhibits PPAR‐γ and leads to cell death by reducing adhesion and inducing anoikis.^323^ In addition, galectin‐3 inhibitors have been shown to be effective in the treatment of thyroid cancer. The mechanism is mainly attributed to the inhibition of anoikis resistance, which is expected to provide a new strategy for inhibiting tumor metastasis.^324^

#### 3.2.2 Targeting CAFs

CAFs have also been proven to be important members involved in promoting tumor metastasis, and targeting CAFs have gradually attracted more attention in the treatment of metastatic tumor.^130^ The activity of CAFs promoting tumor metastasis is mediated by a variety of signaling pathways, especially TGF‐β and EGFR signaling pathways, which may provide targets for anti‐metastasis.^325^, ^326^, ^327^ In a variety of malignancies, including bladder cancer, breast cancer, colorectal cancer, and pancreatic cancer, TGF‐β1 can induce the transformation of resident normal fibroblasts into CAFs and lead to the differential expression of α‐SMA and fibroblast activation protein (FAP) genes (specific markers of CAFs) through the typical TGF‐β signaling pathway.^161^, ^328^, ^329^, ^330^ These data suggest that TGF‐β1 plays a role in promoting CAFs production. 

As the first oral small‐molecule selective inhibitor of TGF‐β1, galunisertib has been proved to have anti‐tumor effects in clinical trials of liver cancer, pancreatic cancer, and glioma.^331^, ^332^, ^333^ In addition, the therapeutic effect of combination gemcitabine with galunisertib is significantly better than gemcitabine monotherapy in advanced pancreatic cancer (combination group 8.9 months vs. monotherapy group 7.1 months).^332^ In a Phase II trial, the median overall survival of galunisertib combined with the multi‐target tyrosine kinase inhibitor (TKI) sorafenib in the treatment of metastatic liver cancer was 18.8 months.^331^ Researchers have also developed a fusion protein, M7824, which blocks both PD‐L1 and TGF‐β signaling pathways; M7824 has shown potent anti‐tumor activity in preclinical studies.^334^ Subsequently, a Phase I clinical study was conducted in advanced lung cancer patients, and M7824 showed tolerable toxicities and preliminary anti‐tumor effects.^335^

In addition, a variety of cytokines secreted by CAFs act as ligands on the janus kinase‐signal transducer and activator of transcription (JAK/STAT) pathway, participate in the activation of this pathway, and promote tumor metastasis.^336^, ^337^, ^338^ For example, CAFs‐derived IL‐6 can activate the STAT3 pathway to promote EMT of tumor cells, thereby facilitating metastasis.^62^, ^339^ Therefore, antagonizing those cytokines or blocking JAK/STAT signaling can inhibit the occurrence of tumor metastasis. The JAK inhibitor ruxolitinib has been shown to inhibit the invasion and migration of breast cancer, lung cancer, and colorectal cancer cells in vivo.^336^, ^340^, ^341^ Two Phase II clinical trials have shown that ruxolitinib combined with gemcitabine improved overall survival in patients with advanced HER2‐negative cancer or advanced pancreatic cancer.^342^ The IL‐6 receptor inhibitor tocilizumab and IL‐6 antagonist siltuximab have been shown to augment the anti‐tumor activity of chemotherapeutic agents in preclinical trials, and further clinical studies are expected.^343^, ^344^

Blocking the activity of CAFs directly can also play an anti‐tumor metastasis role. FAP is one of the markers of activated CAFs, and targeting FAP could transfer activated CAFs into a quiescent state. The monoclonal antibody targeting FAP (sibrutuzumab) and the small molecule inhibitors of FAP (F19 and PT100) were well‐tolerated, but no obvious clinical anti‐tumor effect was observed in Phase I clinical trials, and subsequent structural adjustments may be needed.^345^, ^346^, ^347^

#### 3.2.3 Targeting angiogenesis

Angiogenesis is considered to be one of the components involved in tumor metastasis. A variety of small‐molecule tyrosine kinase inhibitors and monoclonal antibody against angiogenesis have been approved as first‐line or second‐line therapy in a variety of advanced malignancies, including pazopanib targeting vascular endothelial growth factor receptor (VEGFR), imatinib targeting platelet‐derived growth factor receptor (PDGFR), bevacizumab targeting VEGF, and multi‐targeted receptor TKI anlotinib.^348^, ^349^, ^350^, ^351^ In addition, the combined application of existing drugs and the development of new drugs inhibiting angiogenesis are being explored in pre‐clinical and clinical trials.^352^ Several clinical trials of advanced lung cancer have shown that the combination of targeted drugs such as gefitinib, erlotinib, and anlotinib with radiotherapy can enhance the efficacy of anti‐brain metastasis and are expected to be applied in clinical practice.^354^, ^355^, ^356^ There has been a large amount of literature reviewing the therapies targeting angiogenesis, so we will not describe them in detail.

### 3.3 Immunotherapy

#### 3.3.1 Targeting TAMs and Tregs

Regulating tumor immune microenvironment is another crucial therapeutic strategy to interfere with tumor metastasis. There is increasing evidence that TAMs and Tregs in the microenvironment promote tumor development and metastasis.^93^, ^353^ Targeting TAM and Tregs is a promising strategy to modify the immunosuppressive TME and prevent metastasis.

A large number of studies have shown that CC chemokine receptors are essential mediators involved in tumor metastasis.^357^ Among them, CCR1, CCR2, CCR3, and CCR5 promote the recruitment of TAMs in the TME, and CCR4 mainly mediates the recruitment of Tregs, indicating that CC chemokines can be used as potential anti‐tumor pharmacological targets.^82^, ^175^ In recent years, CCR inhibitors have shown strong anti‐tumor effects in preclinical and clinical studies.^358^ For example, in a Phase I clinical study, CCR5 antagonists have demonstrated their ability to inhibit liver metastasis in colon cancer.^359^ A preclinical experiment has shown that mogamulizumab, which antagonizes CCR4, can reduce tumor lung metastasis, and a Phase III clinical trial has demonstrated that antibodies targeting CCR4 are effective in treating patients with cutaneous T lymphoma.^360^ Furthermore, mogamulizuma combined with the immune checkpoint inhibitor nivolumab may provide a new strategy for the treatment of advanced or metastatic solid tumors.^361^ Another monoclonal antibody targeting CCR, AMG 820, also showed preliminary anti‐tumor ability.^362^

Colony‐stimulating factor 1 (CSF1) can enhance macrophage recruitment and activation into a pro‐tumoral TAM phenotype and plays a role in promoting metastasis.^363^, ^364^ The pleiotropic signaling of CSF1 receptor (CSF1R) supports multiple functions of macrophage, including proliferation, differentiation, and migration.^365^ The CSF1/CSF1R axis has received much attention, and approaches targeting the ligands or receptors are currently being clinically developed. Pexidartinib, which target KIT, CSF1R, and Fms‐like tyrosine kinase 3 (FLT3), has been approved by the US Food and Drug Administration (FDA) for the treatment of symptomatic tenosynovial giant cell tumor in adult patients.^366^ Emactuzumab, a humanized monoclonal antibody targeting CSF1/CSF1R, inhibit tumor cell proliferation and metastasis by blocking the activity of CSF1R‐dependent TAMs, suppressing the recruitment of TAMs to the microenvironment, and enhancing the T‐cell infiltration.^367^ However, the anti‐tumor ability of emactuzumab is not evident in a Phase I clinical trial, and further studies are needed.^367^

The CD4+ Treg cell population in the TME is the main cell group leading to the inactivation of effector T cells and is also one of the factors that promote tumor progression and metastasis. Therefore, targeting CD4+ Treg cells directly is a promising strategy to effectively prevent tumor metastasis and progression. IT1208, a defucosylated humanized anti‐CD4 monoclonal antibody, demonstrated tolerable toxicities and encouraging preliminary efficacy in a Phase I clinical trial involving 11 patients with advanced solid tumors.^368^ In addition, a microsatellite‐stable colon cancer patient receiving IT1208 treatment showed increased infiltration of both CD4+ and CD8+ T cells in the tumor and achieved a durable partial response.^368^ These results suggest that Tregs‐targeted drugs, such as IT1208, are expected to provide a new idea for immunotherapy, but further researches are needed.

#### 3.3.2 ICIs

A major hallmark of T‐cell exhaustion is the increased expression of multiple immune checkpoints, such as PD‐1, CTLA‐4, LAG‐3, T‐cell immunoglobulin‐3 (TIM‐3), and T‐cell immunoglobulin and ITIM domain (TIGIT).^369^, ^370^, ^371^ Immune checkpoints have distinct ligands and inhibit anti‐tumor activity of T cell through multiple mechanisms; T cell function decreases with the increase of immune checkpoint expression.^373^ Recent advances in cancer immunotherapy have shown that ICIs, including inhibitors of PD‐1, PD‐L1, CTLA‐4, LAG‐3, TIM‐3, and TIGIT, can improve the clinical response and survival of patients with a broad spectrum of metastatic cancers, such as melanoma, non‐small lung cancer, and renal cell cancer (Table 2).^13^, ^369^, ^371^, ^374^, ^375^, ^376^ ICIs limit the inhibitory pathway of T cells and stimulate the activation of effector T cells to enhance the anti‐tumor immune response. So far, at least eight different ICIs have been approved by the US FDA for the treatment of more than a dozen different cancers, including melanoma, kidney cancer, lymphoma, colorectal cancer, lung cancer, head and neck carcinoma, liver cancer, and sarcoma.^377^

**TABLE 2:** Important immune checkpoint inhibitors under clinical trial and on the market

**Target**	**Phase I**	**Phase II**	**Phase III**	**On the market**
PD‐1	CS1003	MGA012	Cemiplimab	Nivolumab
	ZKAB001	GLS‐010	Camrelizumab	Pembrolizumab
	MK‐3475	Balstilimab	HLX10	Sintilimab
	PF‐06801591	SG001	Penpulimab	Tislelizumab
	AGEN1777	BGB A317	REGN2810	Dostarlimab
	609A	Retifanlimab	Spartalizumab	Toripalimab
	AMP‐224	Zimberelimab	JS001	
			PF‐06801591	
			INCMGA00012	
			BCD‐100	
			IBI308	
			JNJ‐63723283	
PD‐L1	LY3300054	STI‐3031	ZKAB001	Atezolizumab
	KN035	CS1001	SHR‐1316	Avelumab
	BMS‐936559	BGB‐A333		Durvalumab
	HLX20	LP002		Camrelizumab
	MSB2311	Bintrafusp alfa		
	BCD‐135			
CTLA4	CS1002	Quavonlimab	Tremelimumab	Ipilimumab
	BCD‐145	AGEN1884	MDX‐010	
	ADU‐1604	BCD‐217		
	ONC‐392	BMS‐986218		
	ADG126	CP 675,206		
	ADG116	IBI310		
TIGIT	JS006	EOS‐448	BGB‐A1217	
	ASP8374	Ociperlimab	Tiragolumab	
	COM902	BMS‐986207		
	AZD2936	Etigilimab		
	EOS‐448			
	IBI939			
LAG‐3	REGN3767	IMP321		
	TSR‐033	Relatlimab		
	Sym022	LAG525		
		INCAGN02385		
TIM‐3	Sym023	TSR‐022		
	INCAGN2390	MBG453		
	LY3321367	BMS‐986258		
	SHR‐1702	INCAGN02390		
		Cobolimab		
VISTA	JNJ‐61610588			
B7‐H3	MGD009		Enoblituzumab	
Dual PD‐1/PD‐L1		IBI318		
Dual PD‐1/TIGIT		AZD2936		
Dual PD‐1/TIM‐3	RO7121661	AZD7789		
Dual PD‐1/LAG‐3	RO7247669		MGD013	
	MGD013			
Dual PD‐1/VEGF		AK112		
Dual PD‐1/CTLA4		AK104		
		BCD‐217		
Dual PD‐L1/LAG‐3	FS118	RO7247669		
	IBI323			
Dual PD‐L1/TIM‐3	LY3415244	RO7121661		
Dual PD‐L1/4‐1BB		ABL503		
Dual PD‐L1/VISTA	CA‐170			
Dual PD‐L1/TGF‐β	Y101D	SHR1701	M7824	

*Note*: All the data source information is from ClinicalTrials.gov.

Abbreviations: CTLA4, cytotoxic lymphocyte‐associated protein 4; LAG‐3, lymphocyte activation gene 3; PD‐1, programmed cell death 1; PD‐L1, programmed death ligand‐1; TIGIT, T cell immunoglobulin and ITIM domain; TIM‐3, T‐cell immunoglobulin‐3.

Among them, immune checkpoint therapy‐targeting PD‐1/PD‐L1 pathway has achieved remarkable success in various types of tumors. For example, the most dramatic effects have been observed in metastatic melanoma, a malignancy that only slightly responds to conventional chemotherapy, and the historical average survival time of metastatic melanoma patients is less than 1 year.^378^ Surprisingly, when combined with anti‐CTLA4 and anti‐PD‐1 therapy, nearly 60% of the patients can achieve radiographic responses, with a median survival time of more than 3 years.^379^, ^380^ In a Phase II trial, 42 patients with brain metastases from non–small‐cell lung cancer (NSCLC) were recruited to receive PD‐1 targeting inhibitor pembrolizumab, 11 of whom responded, and the median follow‐up time was 8.3 months.^381^ In addition, clinical studies have shown that the PD‐L1 inhibitor atezolizumab can significantly improve the prognosis of patients with advanced or metastatic solid tumors.^382^ Pembrolizumab and atezolizumab have been approved as first‐line or second‐line therapies for some patients with advanced or metastatic solid tumors, such as NSCLC, kidney cancer, breast cancer, and melanoma.^381^, ^382^, ^383^

However, only a limited number of patients can benefit from immunotherapy, and some patients who initially respond to immunotherapy may eventually relapse and progress.^384^ Therefore, a large number of pre‐clinical and clinical studies have investigated the combination of immunotherapy with other therapies, including chemotherapy, radiotherapy, or targeted therapy, as well as the combination of ICIs targeting different immune checkpoints to overcome the dilemma. To date, many therapies combined with immunotherapy and chemotherapy or anti‐angiogenic drugs have been recommended as first‐line treatments for multiple solid tumors.^385^, ^386^ For example, in patients with squamous NSCLC, the addition of pembrolizumab to chemotherapy (carboplatin plus paclitaxel or nab‐paclitaxel) result in significantly better overall survival and progression‐free survival than chemotherapy alone.^387^ As a result, the combination of pembrolizumab with chemotherapy is recommended as the first‐line treatment for patients with advanced squamous NSCLC.^388^ In advanced renal cell carcinoma, the FDA approved pembrolizumab combined with lenvatinib or axitinib as the first‐line treatment.^389^, ^391^ Among them, pembrolizumab combined with axitinib is the first anti‐PD‐1 antibody plus targeted drug combination therapy approved by the FDA.^390^, ^391^ Moreover, it has been reported that radiotherapy can improve the response of patients with metastatic NSCLC to immunotherapy.^392^ Overall, a broad range of immunotherapy‐based combination therapies have been approved for the treatment of cancer, and a large number of preclinical studies and clinical trials are ongoing to explore more possibilities for combination therapies in the treatment of metastatic cancer.

### 3.4 Others

In recent years, due to the abundance of natural herbal compounds and the diversity of their chemical compositions, they have received more and more attention as anti‐tumor drugs. Triptolide, anthocyanidins, and gigantol have been confirmed to inhibit the proliferation and invasion of NSCLC cells, glioblastoma cells, and bladder cancer cells in vivo and in vitro.^393^, ^394^, ^395^ Studies have found that the anti‐tumor effects of certain compounds are mainly achieved by inhibiting EMT.^394^, ^395^ In addition, the chemical compound DZ‐50 and the natural product curcumol can inhibit tumor metastasis by weakening the resistance of tumor cells to anoikis.^396^, ^397^ However, their anti‐tumor effects need to be confirmed by further clinical studies.

The failure of metastatic cancer treatment may be partly due to the inability of drugs to persist in the blood or lymph and the inability of drugs to cross certain natural barriers. For example, for patients with metastatic brain cancer, the difficulty in treatment is that most anti‐tumor drugs cannot cross the blood‐brain barrier. The emergence of nanomedicine has brought revolutionary updates to tumor treatment. Due to the small molecular weight of nanometers and the ability to carry specific pharmacophores to distant metastatic regions, nano‐related chemotherapy and nano‐related immunotherapy have demonstrated outstanding potential in tackling metastatic cancer.^398^, ^399^

The combination of different nano‐polymer materials (such as hydrogels, micelles, and nanoparticles) and chemotherapeutic drugs, small molecule inhibitors, or immunotherapy drugs, has successfully improved the control rate of metastatic tumor.^400^, ^401^, ^402^, ^403^ For example, losartan‐loaded injectable peptide hydrogel can inhibit the formation of CAFs collagen, thereby increasing the concentration of adriamycin at the metastatic sites and killing tumor cells.^400^ Succinobucol is a small molecule inhibitor targeting vascular cell adhesion molecule‐1, which is believed to be able to reverse lung metastasis, but the results in in vivo experiments are not satisfactory. Then, He et al. successfully designed succinobucol on ph‐responsive wormlike micelles as a novel nanomedicine. Both in vivo and in vitro experiments demonstrated that succinobucol could be successfully delivered to the site of metastasis through ph‐responsive wormlike micelles, thus improving treatment efficiency.^401^ In addition, nanotechnology can enhance the anti‐tumor effect of immunotherapy by rebuilding the tumor immune microenvironment, enhancing the anti‐tumor effect of immune cells, enhancing the effectiveness of anti‐tumor cytokines, and awakening tumor cells to release antigens.^398^, ^399^ Moreover, nanotechnology may reduce the side effects of immunotherapy.

## 4 CONCLUSIONS AND FUTURE PERSPECTIVES

Metastasis accounts for the great majority of cancer‐related deaths, which is the result of the interaction between tumor cells and TME components. In contrast to numerous discoveries that have revealed the detailed mechanisms leading to the formation of primary tumor, the biological underpinnings of metastatic disease are still poorly understood. However, some progress has been made in elucidating the cellular and molecular mechanisms that drive cancer metastasis over the past decades.

Despite the diversity of tumor types, the mechanisms of metastasis have some commonalities. The invasion–metastasis cascade involves angiogenesis, detachment of metastatic cells from the primary tumor, invasion through the BM and ECM rounding tumor, intravasation of the metastatic cells into the blood and lymphatic vessels and survival during hematogenous transit, adhesion of these cells to the endothelium of capillaries, extravasation through vascular walls into the parenchyma of distant tissues, and colonization at the target organ site.^17^, ^23^, ^404^ The EMT and anoikis resistance of tumor cells are the main forces to promote the metastasis cascade, and multiple components in TME are inducers to ensure the success of metastasis by reprogramming the pre‐metastasis niche and promoting metastatic cell growth.^21^, ^35^, ^325^ EMT modulates cell‐to‐cell or cell‐to‐matrix adhesion and allows cells to increase their migration and invasion capabilities by forming invasive protrusions.^405^ Metastatic cells develop an ability to resist anoikis, enabling them to adapt and survive when detached from the ECM or in the absence of the ECM.^19^ The interactions between tumor and ECM components are essential for EMT and acquisition of tumor invasive abilities, and TME is also closely involved with all the processes of metastasis.^69^ As presented earlier, immune cells (e.g., TAMs, Treg, and neutrophil), stromal cells (e.g., CAFs, MSCs, and TECs), chemokines, cytokines, and growth factors in TME, are involved in a complex crosstalk with tumor cells that affects tumor growth and metastasis.^69^, ^79^, ^138^, ^406^ Furthermore, recent studies have emphasized the role of autophagy, lipid metabolism, and lncRNA in tumor metastasis.^212^, ^240^, ^255^

Those discoveries have brought new insight into our understanding of cancer metastasis. For most patients, due to the insidious tumor‐related symptoms, distant metastasis has already appeared at the time of diagnosis.^1^ In addition to the three cornerstones of tumor treatment, surgery, chemotherapy, and radiotherapy, novel treatment approaches, including targeted therapy and immunotherapy, have been established in patients with metastatic cancer. According to the chronological order and biological characteristics of metastasis, intervention strategies can be roughly classified into four types: early prevention, blocking metastasis‐specific pathways, reducing angiogenesis, and enhancing anti‐tumor immunity. For low‐grade early malignancies, preoperative neoadjuvant chemotherapy and postoperative adjuvant chemotherapy can prevent and reduce the probability of recurrence and metastasis to a certain extent.^298^, ^301^ For advanced or metastatic tumors, targeted therapies, especially drugs targeting metastasis‐specific pathways and angiogenesis have been approved for the treatment of most types of tumors.^15^ What is more exciting is that immunotherapy, especially ICIs, is revolutionizing the treatment of malignancies and has demonstrated their potential as “cancer terminators.” The advent of immunotherapy and combination therapies based on immunotherapy has provided more options for patients with metastatic cancer. Despite many challenges, we hold an optimistic view of the future prospect of immunotherapy.

In summary, this review provided our current understanding of the mechanisms that underlie the dissemination and metastatic outgrowth of cancer cells. As presented above, although many critical obstacles are still lying ahead, tumor metastasis‐related pathways and TME represent novel and attractive directions, which may change the landscape of cancer therapy in the future. However, further researches and more endeavors in this area are needed.

## CONFLICT OF INTEREST

We declare that we do not have any commercial or associative interest that represents a conflict of interest in connection with the work submitted.

## ETHICS APPROVAL

Not applicable.

## AUTHOR CONTRIBUTIONS

ML and YJ: data curation, formal analysis, and writin—goriginal draft. BX: data curation and formal analysis. XZ: conceptualization, supervision, validation, and writing—review editing. All authors read and approved the final manuscript.

## Data Availability

Not applicable.
